# Metals Are Important Contact Sensitizers: An Experience from Lithuania

**DOI:** 10.1155/2017/3964045

**Published:** 2017-03-16

**Authors:** Kotryna Linauskienė, Laura Malinauskienė, Audra Blažienė

**Affiliations:** Clinic of Infectious and Chest Diseases, Dermatovenereology and Allergology, Centre of Pulmonology and Allergology, Vilnius University, Vilnius, Lithuania

## Abstract

*Background.* Metals are very frequent sensitizers causing contact allergy and allergic contact dermatitis worldwide; up-to-date data based on patch test results has proved useful for the identification of a problem.* Objectives.* In this retrospective study prevalence of contact allergy to metals (nickel, chromium, palladium, gold, cobalt, and titanium) in Lithuania is analysed.* Patients/Methods.* Clinical and patch test data of 546 patients patch tested in 2014–2016, in Vilnius University Hospital Santariskiu Klinikos, was analysed and compared with previously published data.* Results.* Almost third of tested patients (29.56%) were sensitized to nickel. Younger women were more often sensitized to nickel than older ones (36% versus 22.8%, *p* = 0.0011). Women were significantly more often sensitized to nickel than men (33% versus 6.1%, *p* < 0.0001). Younger patients were more often sensitized to cobalt (11.6% versus 5.7%, *p* = 0.0183). Sensitization to cobalt was related to sensitization to nickel (*p* < 0.0001). Face dermatitis and oral discomfort were related to gold allergy (28% versus 6.9% dermatitis of other parts, *p* < 0.0001). Older patients were patch test positive to gold(I) sodium thiosulfate statistically significantly more often than younger ones (44.44% versus 21.21%, *p* = 0.0281).* Conclusions.* Nickel, gold, cobalt, and chromium are leading metal sensitizers in Lithuania. Cobalt sensitization is often accompanied by sensitization to nickel. Sensitivity rate to palladium and nickel indicates possible cross-reactivity. No sensitization to titanium was found.

## 1. Introduction

Allergic contact dermatitis (ACD) is one of the most common skin diseases [1], although the precise molecular mechanism still remains unknown [2].

Contact with metals, such as gold (Au), nickel (Ni), chromium (Cr), cobalt (Co), titanium (Ti), and palladium (Pd) is very frequent in daily life as metal alloys are widely used in jewelry, toys, coins, household appliances, dental materials, or glasses.

The diagnosis of ACD is based on clinical history and patch testing. Patch testing is a very effective mean to investigate the cause of contact allergy. The most important metal allergens (Ni, Co, and Cr) are included in “baseline series” that are routinely applied to most of patients with suspected ACD, as a general screening test [3]. According to patient's complaints and for more precise evaluation of sensitization to metals, dental screening series and/or metal series are used. The aim of this study was to examine the prevalence of contact allergy to metals in patients with suspected ACD at Vilnius University Hospital Santariskiu Klinikos and to compare compiled patch test data with previously published results from other countries.

## 2. Methods

### 2.1. Patients

In this retrospective study, we analysed the patch tests results of the 546 patients referred to our clinic in 2014–2016 with suspected ACD. Five hundred and four patients of them were tested with European baseline series: 87.7% (489/504) were women and 12.9% (65/504) men, with age average 42 years. Sensitization frequencies to nickel, cobalt, and chromium were analysed according to European baseline series patch testing results. Primarily allergy to metals was suspected in 87 patients who had oral symptoms or were dentists. These patients were patch tested only with dental screening series or metal series, 91.9% (80/87) were women, and 8.1% (7/87) were men (average age 48 years). Sensitization frequencies to gold and palladium were analysed using dental screening and metal series patch testing results.

### 2.2. Patch Testing

Patch testing was performed and scored following the European Society of Contact Dermatitis guideline for diagnostic patch testing [8]. The haptens (European baseline, dental screening, and metal series) were provided by Chemotechnique Diagnostics (Vellinge, Sweden). Finn Chambers (ø 8 mm, Epitest Ltd, Tuusula, Finland) on Scanpor tape (Norgesplaster A/S, Vennesla, Norway) were used for patch testing. Fifteen *μ*L of test solution was applied with a micropipette to the filter paper discs in the chamber or 20 mg of test preparation in the petrolatum to each test chamber [9]. The chambers were applied and left on the back for 48 hours and the readings were done on day 3 (D3), D4, and D7 by allergologist trained to perform patch testing. For the patch test analysis, the maximal patch test reactions from either D3/D4 or D7 were considered as an outcome. Reactions “+” to “+++” were classified as positive, and negative and irritant and doubtful reactions as nonpositive.

### 2.3. Statistical Analysis

*λ* ^2^ or Fisher's exact test was used where appropriate (if *n* ≤ 5). All results were expressed as odds ratios, and the threshold for statistical significance was predefined as a *p* value of <0.05. Data were analysed with Microsoft® Excel® MSO version for Windows™.

## 3. Results

### 3.1. Patient Characteristics

The demographics of the patch-tested patients are summarized in Table 1. The most common locations of dermatitis were face and hands. Statistically significantly (*p* < 0.0001) more patients tested with dental screening and metal series had complaints of face dermatitis or oral problems. Out of 546 tested patients, 345 (63.3%) had at least one positive patch test reaction.

### 3.2. Sensitization Frequencies

The positive patch test results for tested metals are shown in Table 2. 29.6% of patch-tested patients were sensitized to nickel. Women younger than 40 years were patch test positive to nickel sulphate statistically significantly more often than older ones (36.0% versus 22.8%, *p* = 0.0011). Women were significantly more often patch test positive to nickel than men (33.0% versus 6.1%, *p* < 0.0001). Sensitization rate to cobalt chloride was 8.7%. Cobalt chloride was more often patch test positive in women than in men (9.1% versus 6.1%, *p* = 0.4) but without statistical relevance. Patients younger than 40 years were statistically significantly more often sensitized to cobalt (11.6% versus 5.7%, *p* = 0.0183). Sensitization to cobalt was statistically significantly related to sensitization to nickel (70.5% of patients sensitive to cobalt were sensitized to nickel also, *p* < 0.0001). Sensitization rate to chromium was 6.5%. Women and men were sensitized to potassium dichromate almost equally (6.2% versus 9.2%, *p* = 0.34) and age had no influence on this sensitization (6.9% versus 6.1%, *p* = 0.69).

Forty percent of patients tested with the dental screening series and 26.0% tested with metal series were sensitized to gold(I) sodium thiosulfate 2.0%. Face dermatitis and oral discomfort were related to gold allergy (28.0% versus 6.9% dermatitis of other parts, *p* < 0.0001). Men and women were similarly sensitized to gold (36.3% versus 28.6%, *p* = 0.68). Patients older than 40 years were patch test positive to gold(I) sodium thiosulfate statistically significantly more often than younger ones (44.4% versus 21.2%, *p* = 0.0281).

Palladium(II) chloride and sodium tetrachloropalladate(II) hydrate were patch test positive in 18.0% and 7.0% patients tested with the dental screening series and in 16.7% and 8.5% patients tested with metal series. No men were patch test positive to palladium salts. No statistical significant relation with patients age was found.

## 4. Discussion

Our analysis revealed that gold and nickel are the most prevalent patch test positive metals. Women especially under the age of 40 years are more often sensitized to nickel comparing to men. This could be attributed to patterns of nickel exposure, early ear piercing, and contact with nickel releasing jewelry. The European Directive regulating nickel release from metal items was approved in Lithuania only in 2002 [10, 11]. In 2016 we have higher or similar sensitization rate to nickel sulphate than European countries had in 2005-2006 (Table 2). High sensitivity to nickel still remains in other European countries, but this is declining. Data from Finland and Poland Contact allergy centres showed sensitization rates to nickel of 21.3% and 24.3%, respectively, in 2007-2008 [12, 13]. According to Thyssen et al. the decrease of nickel sensitization in Denmark started 16 years after the nickel regulation and mainly depended on delayed ear piercing time [14]. So it could be speculated that sensitization to nickel in Lithuania will start to decrease in 2020 and the shifting of nickel sensitization to the older age (>40 years) group can be expected in the future.

Gold allergy was detected in more than third of the tested patients. Almost half of them (44.2%) had golden dental crowns for a long time. This mostly explains the high rate of positive patch test reactions especially in older patients. Compared to other countries Lithuania is leading in gold sensitization (Table 2). It is often very difficult to find clinical relevance of positive reaction [15]. It was an overrepresentation of face dermatitis in the group of patients with gold allergy although we did not find direct causal relationship to gold allergy.

It has been postulated that nickel allergy may predispose to cobalt allergy [13]. Monosensitization to cobalt is rare. However high cobalt contact sensitization is observed in countries centres where occupational dermatitis is diagnosed and treated, for example, in Finland (9.5%) and Poland (12.2%), but also Austria (13.6%) and Switzerland (9.9%). Often, exposure to cobalt remains unclear in patients with cobalt sensitization [3, 12]. Studies show that only a small part of inexpensive jewelry released cobalt when assessed with the available cobalt spot test [16]. It is known that cobalt ions release from both bright and dark and from both expensive and inexpensive jewelry [17]. So maybe this could be an explanation outside an occupational exposure.

Sensitization rate to chromium in Lithuania is 6.5% and with no difference in men and women. In 6-year period sensitization remains stable (Table 2) and nearly equal to Poland's sensitization (6.9%) in year 2007. However, chromium is important contact sensitizer that proves even higher sensitization rate in Austria (9.5%), while lowest sensitization rates are in Denmark (1.7%), The Netherlands (2.9%), and United Kingdom (2.3%) [3, 12] and this could be associated with the European commission directive on hexavalent chromium implemented in June 2002 (Lithuania implemented directive in 2006) [11]. Nowadays leather items produced using chromium salts are considered to be the main source of sensitization (probably) and elicitation of ACD although we were unable to show this in our patients.

Our results on sensitization to palladium chloride are opposite to the European multicentre study where they found tetrachloropalladate to be better in pin-pointing sensitization to palladium than palladium chloride. However population tested was much bigger than in our study. There are publications analysing the correlation between palladium and nickel cross- or cosensitization. In our patients, patch tested with the dental screening series, we noticed that 76.9% (10/13) of palladium positive patients were sensitized to nickel. This confirms previously published data on nickel and palladium cross-reactivity. Although the role of palladium and nickel sensitization in oral diseases and ACD is not fully understood, palladium as a causative agent of contact allergy is important and according to Muris et al. palladium sensitization is associated with dental crowns, skin reactivity to other metals, oral lichenoid reactions, xerostomia, and metal taste, but not with stomatitis or oral burning sensation [18].

In our study, we had no patch test positive reaction to any of the five titanium salts present in the metal series. This might be because of relatively small number of patients tested.

In conclusion, this is an up-to-date view on the prevalence of contact allergy to the main heavy metals in Lithuania. Nickel, gold, cobalt, and chromium are leading metal sensitizers in our study. Cobalt sensitization is often accompanied by sensitization to nickel. No sensitization to titanium was found. When comparing data with other European countries, it seems that we had higher prevalence of metal allergy and mostly this can be attributed to different exposure patterns.

## Figures and Tables

**Table 1:** MOAHLFAP characteristics of the tested population in 2014–2016.

Characteristics	European baseline series (*N* 504)				Dental screening and metal series (*N* 87)
	Number of positive reactions *n* (%)	Nickel *n*/*N* (%)	Cobalt *n*/*N* (%)	Chromium *n*/*N* (%)	Number of positive reactions *n* (%)
Male	65 (12.9)	4/65 (6.2)	4/65 (6.2)	6/65 (9.2)	7 (4.0)
Occupational	0 (0)	0 (0)	0 (0)	0 (0)	0 (0)
Atopic dermatitis	118 (23.4)	31/118 (26.3)	12/118 (10.2)	12/118 (10.2)	9 (8.0)
Hand	156 (30.7)	46/156 (29.5)	19/156 (12.2)	10/156 (6.4)	24 (20.0)
Leg	24 (5.4)	6/24 (25.0)	2/24 (8.3)	2/24 (8.3)	3 (2.0)
Face	232 (47.3)^*∗*^	65/232 (28.0)	16/232 (6.9)	10/232 (4.3)	65 (78.0)^*∗*^
Age > 40 years	246 (48.8)	56/246 (22.8)	14/246 (4.9)	15/246 (6.1)	58 (66.0)
Positivity rate (≥1 positive reaction)	288 (57.1)	149/504 (29.6)	44/504 (8.7)	33/504 (6.6)	61 (66.0)

*N*: total number of tested patients.

*n*: number of positive reactions.

^**∗**^ *p* < 0.0001.

**Table 2:** Test results in comparison with other previously published data.

Haptens	Lithuania 2014–2016	Lithuania 2010–2012 [4]	Sweden 2010–2012 [4]	The European baseline series in 10 European countries 2005-2006 [5]				Korea 2004–2011 [6]	European multicentre study [7]
				Western (United Kingdom)	Southern (Spain, Italy)	Central (Netherlands, Switzerland, Austria, Germany)	Northeast (Finland, Lithuania, Poland)		
	% (*n*/*N*)	% (*n*/*N*)	% (*n*/*N*)	% (*n*/*N*)	% (*n*/*N*)	% (*n*/*N*)	% (*n*/*N*)	% (*n*/*N*)	% (*n*/*N*)
Nickel sulphate	29.6 (149/504)	25.7 (55/214)	18.9 (81/428)	20.8 (1752/8468)	24.5 (660/2666)	19.7 (1056/5708)	22.4 (348/1585)	25.0 (11/44)^&^	25.2 (228/906)^&^
Cobalt(II) chloride hexahydrate	8.7 (44/504)	7.5 (16/214)	6.3 (27/428)	6.2 (522/8498)	6.8 (178/2616)	7.2 (398/5730)	8.8 (137/1596)	15.9 (7/44)^&^	—
Potassium dichromate	6.6 (33/504)	6.1 (13/214)	2.8 (12/428)	2.4 (211/8537)	4.5 (119/2666)	5.9 (347/5737)	5.3 (86/1606)	22.7 (10/44)^&^	—
Gold(I) sodium thiosulphate	35.6 (31/87)^&^	23.8^∧^	13.6^∧^	—	—	—	—	25.0 (11/44)^&^	—
Palladium(II) chloride	14.9 (13/87)^&^	—	—	—	—	—	—	6.8 (3/44)^&^	24.3 (220/906)^&^

*n*: positive test reactions.

*N*: total tests.

^&^Population tested with dental screening or metal series.

^∧^Consecutive patients tested with European baseline series with added gold(I) sodium thiosulphate.
